# Large congenital cervical mass in a neonate: prenatal diagnosis and postnatal management of teratoma: a case report

**DOI:** 10.1186/s13256-024-04535-x

**Published:** 2024-05-17

**Authors:** Fateme Ziyaee, Mehdi Forooghi, Bita Geramizadeh, Hossein Fatemian, Mehdi Ghasemian

**Affiliations:** 1 Department of Pediatric Surgery, Shiraz University of Medical Sciences, Shiraz, Iran; 2 School of Medicine, Shiraz University of Medical Sciences, Shiraz, Iran; 3 Shiraz Transplant Research Center (STRC), Shiraz University of Medical Sciences, Shiraz, Iran; 4 Department of Pathology, Shiraz University of Medical Sciences, Shiraz, Iran; 5 Department of Pediatric Gastroenterology, Shiraz University of Medical Sciences, Shiraz, Iran

**Keywords:** Case report, Cervical teratoma, Neonatal airway obstruction, Surgical resection

## Abstract

**Introduction:**

Cervical teratomas are rare congenital neoplasms that can cause neonatal airway obstruction if large.

**Case presentation:**

The female Persian neonate displayed respiratory distress at birth, with a 7 cm × 8 cm cystic solid mass identified on the left side of the neck. Antenatal ultrasonography revealed polyhydramnios. Despite initial stabilization, the infant required intubation and mechanical ventilation due to persistent respiratory distress. Imaging confirmed a cystic mass compressing the trachea, ruling out cystic hygroma. Surgical resection on postnatal day 17 revealed a 10 cm × 10 cm solid cystic structure, histologically identified as an immature teratoma.

**Conclusion:**

Despite risks of poor fetal and postnatal outcome from large cervical teratomas, early surgical resection after airway stabilization can result in recovery. Proper multidisciplinary management of respiratory distress from such tumors is paramount.

## Introduction

Cervical teratomas are rare congenital germ cell tumours originating from all three germ layers that can potentially obstruct the airway. While the incidence is estimated at 1 in 20,000–40,000 live births, these tumours represent a diagnostic and management challenge due to risks of respiratory compromise and mortality, which have been reported between 3 and 34% depending on the degree of airway involvement at presentation. [1–4] While prenatal diagnosis allows anticipation of potential airway issues in about 20% of cases, unexpected postnatal presentation remains an area of concern. [5, 6]. We present a case of a neonate with a large cervical teratoma detected on prenatal ultrasound who developed respiratory distress shortly after birth requiring intubation and mechanical ventilation. Despite risks of morbidity and mortality from tumors of this size, early surgical management following airway stabilization resulted in favourable outcome. Presenting details of complex cases adheres to reporting guidelines, which aim to improve transparency and optimize care of similar clinical situations. This manuscript was prepared following the CARE guidelines for case reports. (https://www.care-statement.org)

## Case presentation

**Fig. 1:**
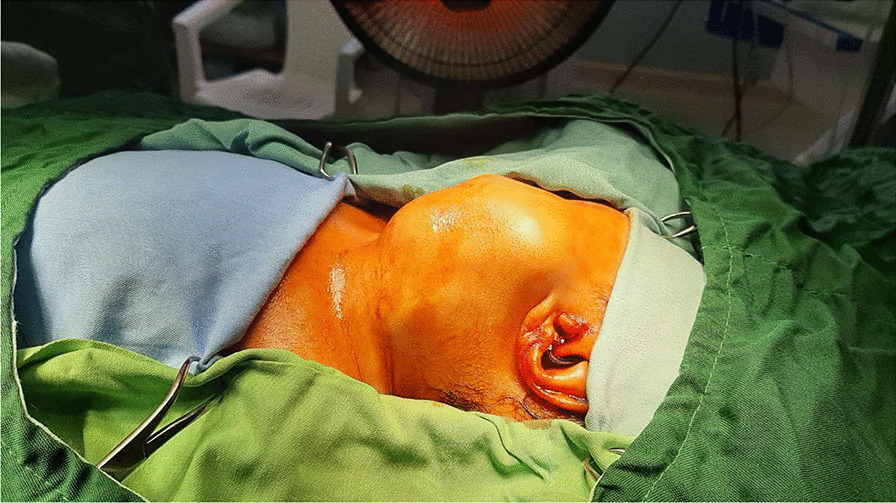
Photograph of neck mass in neonate

A 34-year-old female gravida 4, para 3 delivered a 2900 g female child by spontaneous, uncomplicated vaginal delivery at 36 weeks of gestation. At delivery, a large cervical mass was obvious on the left side, which was cystic solid, measuring about 7 cm × 8 cm on examination (Fig. 1). The child had a respiratory rate of 40, a pulse rate of 120, an oxygen saturation of 56%, and a primary APGAR score of 9. A few seconds postdelivery she developed apnea which was resolved by stimulation and positive pressure ventilation (PPV) for 2 min and the oxygen saturation reached 90%. Based on an ultrasonographic study performed antenatally, the amniotic fluid index was reported to be more than 40 suggestive of polyhydramnios; therefore, the delivery was done with an attendant neonatal team at the delivery room. A neck ultrasonography was requested in which a neck mass with a pressure effect over the trachea was seen.

**Fig. 2:**
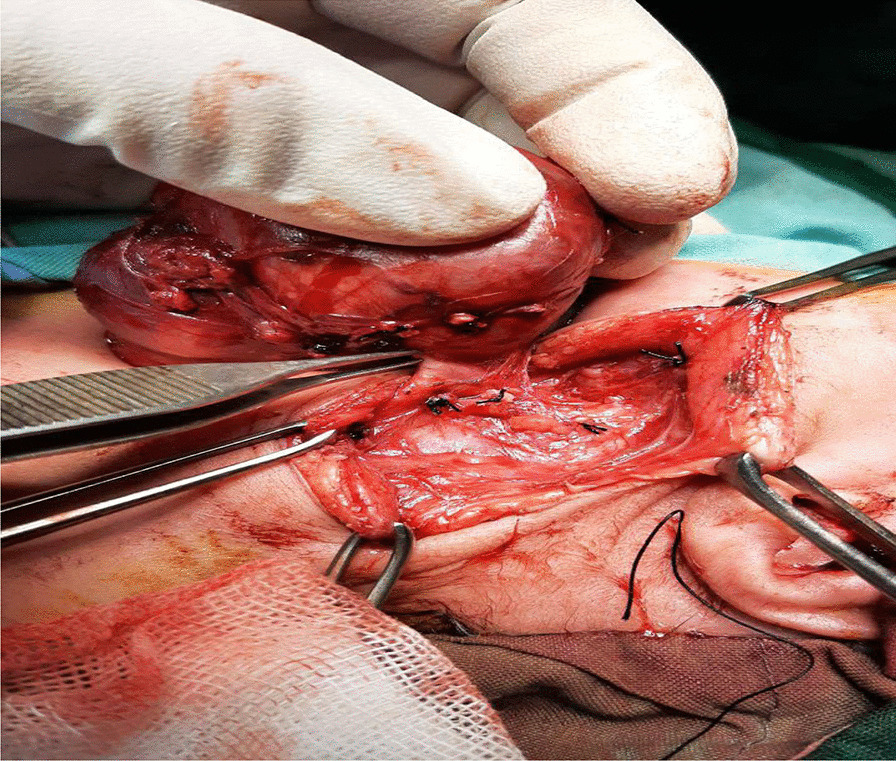
Intraoperative photograph during resection of cervical mass

**Fig. 3:**
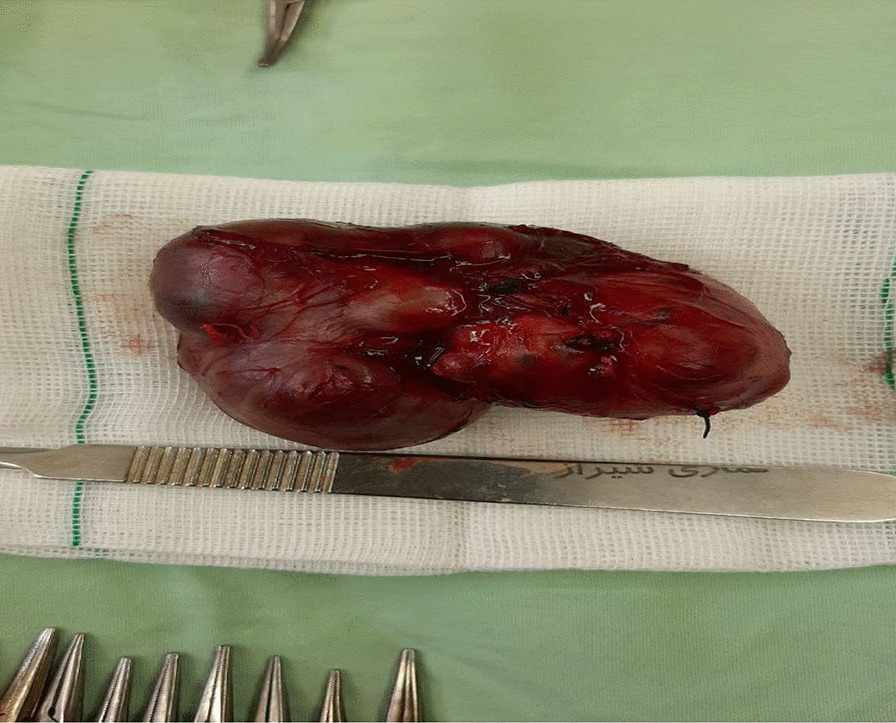
Photograph of excised cervical mass

**Fig. 4:**
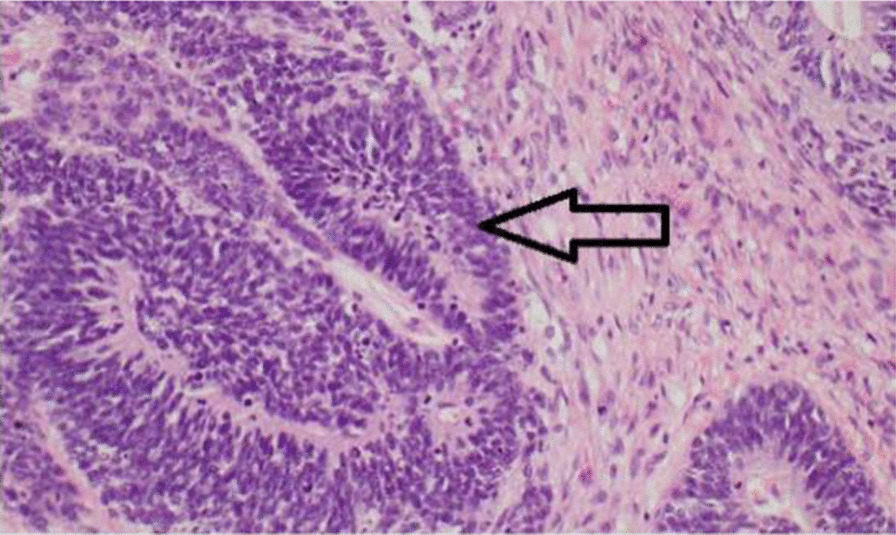
Pathology slide showing components of immature teratoma, neuroepithelial part, indicated by the arrow. (H&E, 250×)

Then she was transferred to the neonatal intensive care unit with supplementary oxygen therapy adfministered over a hood at a rate of 10 L/min. The patient developed respiratory distress again and the oxygen saturation fell below 60%. After a failed trial of CPAP, the child was intubated and mechanically ventilated. With an impression of cystic hygroma with internal bleeding, a neck CT scan with intravenous contrast study was requested containing CT scan report, ruling out cystic hygroma. Mass biopsy under the guidance of ultrasound and then pathologic examination was not suggestive of cervical neuroblastoma, therefore, she was taken to the operating room. Figure 2 shows an intraoperative photograph during resection of the mass. The neck was positioned in right side rotation and was entered through a transverse incision on the left side. Intraoperative finding was a 10 cm × 10 cm solod cystic structure extending from carotid bifurcation to the chest cleft. Figures 3 and 4 show photographs of the excised mass and pathology slide, respectively.

## Discussion

This case describes a rare large cervical teratoma found at delivery in a newborn. Cervical teratomas are uncommon congenital tumors that can compress the airway [1]. Prenatal detection occurs in 20% of cases and aids management by evaluating airway involvement and determining delivery approach [2, 5].

In this case, challenges included late identification of the mass perinatally and respiratory compromise in the newborn necessitating intubation. Fetal MRI can better define airway compression but was not performed [3]. Securing the airway through intubation was crucial given literature linking tumor size and respiratory distress to mortality [4]. Surgical resection is standard treatment [6, 7] and pathology in this case aligned with descriptions of immature teratoma. While prenatal diagnosis allows optimized delivery planning, multidisciplinary care including airway stabilization successfully managed this high-risk presentation.

In conclusion, this case highlights the importance of timely diagnosis and coordinated neonatal resuscitation for large cervical teratomas. With informed consent from the guardian, it demonstrates lessons learned.

## Conclusion

Large cervical teratomas require a multidisciplinary approach including securing the airway.

## Data Availability

The datasets used in this study are available from the corresponding author upon reasonable request. All relevant data supporting the conclusions of this case report are included within the manuscript.
